# Gene Expression Analysis of the Irrigation Solution Samples Collected during Vitrectomy for Idiopathic Epiretinal Membrane

**DOI:** 10.1371/journal.pone.0164355

**Published:** 2016-10-13

**Authors:** Sayaka Myojin, Takeru Yoshimura, Shigeo Yoshida, Atsunobu Takeda, Yusuke Murakami, Yoichi Kawano, Yuji Oshima, Tatsuro Ishibashi, Koh-Hei Sonoda

**Affiliations:** 1 Department of Ophthalmology, Kyushu University, 3-1-1 Maidashi, Higashi-ku, Fukuoka 812-8582, Japan; 2 Section of Ophthalmology, Department of Medicine, Fukuoka Dental College, 2-15-1 Tamura, Sawara-ku, Fukuoka 814-0193, Japan; Massachusetts Eye & Ear Infirmary, Harvard Medical School, UNITED STATES

## Abstract

**Purpose:**

The analysis of gene expression in idiopathic epiretinal membranes (iERMs) may help elucidate ERM formation and its pathology. Here, we conducted a case-control study, in order to determine the expression levels of cytokines and other genes in eyes with macular hole (MH) or iERM.

**Methods:**

Twenty eyes, obtained from seven male and 13 female patients, were included in the study. The average age of the study subjects was 69.1 ± 7.67 years, and 15 eyes had iERM, while five eyes had MH. Irrigation solution samples were collected during vitrectomy, centrifuged, and the levels of cytokine and other mRNAs in the sediment were assessed using real-time PCR. The expression level of 11 cytokine genes, four transcription factor genes, two cytoskeletal genes, and genes encoding two extracellular matrix proteins in eyes with MH or iERM were determined and compared.

**Results:**

The expression levels of interleukin 6 (*IL6*), tumor growth factor B2 (*TGFB2)*, vascular endothelial growth factor A (*VEGFA*), chemokine C-X-C motif ligand 1 (*CXCL1*), v-rel avian reticuloendotheliosis viral oncogene homolog A (*RELA*), glial fibrillary acidic protein (*GFAP*), and tenascin C (*TNC*) were significantly higher in eyes with iERM than in eyes with MH. The expression of these genes was not associated with the preoperative visual acuity of the investigated patients.

**Conclusions:**

The obtained results indicate that real-time PCR analysis of irrigation solution samples collected during vitrectomy can help assess the expression levels of several genes, and that iERM is associated with the expression of pro-inflammatory genes and the genes expressed during angiogenesis and wound healing process (*IL6*, *TGFB2*, *VEGFA*, *CXCL1*, *RELA*, *GFAP*, and *TNC*).

## Introduction

The development of epiretinal membranes (ERMs) can cause visual acuity reduction or vision distortion in the elderly. Idiopathic or secondary ERM can accompany the development of certain diseases such as proliferative vitreoretinopathy, ocular inflammation (*e*.*g*., uveitis), ocular trauma, and postoperative rhegmatogenous retinal detachment, as a response to changes in the vitreous humor [1,2]. ERM is thought to be caused by a fibrocellular proliferation of the inner limiting membrane (ILM) and the subsequent vitreoretinal adhesion and traction [3,4]. Although the precise pathogenesis remains unknown, the measurements of the levels of cytokines and other factors in the solid component of the irrigation solution may aid future studies and help elucidate the role of various proteins in the pathogenesis of ERM. Enzyme-linked immunosorbent assay (ELISA), immunoblotting, and traditional reverse transcription PCR (RT-PCR) have been used to assess the level of cytokines and/or other proteins, such as transcription factors, in the vitreous fluids [5–9].

In this study, we used real-time PCR to examine the expression of genes encoding cytokines and other proteins, and to measure their levels in the solid component of the irrigation solution collected during vitrectomy. ELISA requires the collection of dry vitreous humor, which necessitates the discontinuation of irrigation, and this may lead to a reduction in intraocular pressure. However, our method of choice allows normal vitrectomy, without the need for the discontinuation of the irrigation. Therefore, the mechanisms underlying the idiopathic epiretinal membrane (iERM) pathogenesis can be assessed using real-time PCR and subsequent analysis can be performed comparing the samples from eyes with macular hole (MH) and iERM.

## Materials and Methods

This study was conducted according to the principles outlined in the Declaration of Helsinki and it was approved by the Institutional Review Board of Kyushu University Hospital, Japan. All study participants were informed regarding the potential consequences of the study and written informed consents were obtained from all of them.

### Patients

Fifteen eyes obtained from 15 patients (six males and nine females) with iERM were included in the study. The mean age of the subjects was 71.5 ± 6.59 years. ERM was diagnosed by ophthalmoscopy and spectral-domain optical coherence tomography (OCT) (Cirrus HD-OCT, Carl Zeiss Meditec, Dublin, CA, USA). These patients underwent cataract surgery and vitrectomy at the Kyushu University Hospital, Fukuoka, Japan, between April and October 2013. The eyes of all patients were treated with an intravitreal vital dye Brilliant Blue G and triamcinolone acetonide. Patients with concomitant ocular lesions, such as those that underwent previous intravitreal injection and eye surgery, or suffered from ocular trauma, diabetic retinopathy, uveitis, retinal tears or holes, and retinal artery or vein occlusions, were excluded from the study. Five patients with ILM (one male and four females) who underwent vitrectomy for MH without ERM were included as a control group (mean age, 61.8 ± 5.84 years). One patient with MH had hyperlipidemia and asthma as well, while another patient had Sjögren's syndrome. Additionally, three patients with iERM had hypertension and four patients had heart diseases. Their treatment included warfarin in three of these patients and verapamil in one. The other patients had no systemic diseases, including autoimmune diseases.

### Sample collection

Irrigation solution samples were collected during three-port 25-gauge closed vitrectomies. In eyes with cataract, phacoemulsification and aspiration were performed first, while the vitrectomy was performed after changing the irrigation solution cassette, and these irrigation solution samples were collected immediately after each surgery. Irrigation solution samples were centrifuged at 1,100 ×*g* for 30 min and afterward at 2,000 ×*g* for 10 min. Supernatants were discarded after each centrifugation. The pellets were homogenized in 300 μL of TRIzol reagent (Invitrogen, Thermo Fisher Scientific, Waltham, MA, USA), using Fast Prep System and Lysing Matrix D (FastRNA Green; QBiogene, Illkirch Cedex, France), according to the manufacturer’s instructions. Following the addition of 60 μL of chloroform, samples were centrifuged at 18,000 ×*g* for 15 min at 4°C, and the aqueous phase was collected for a subsequent isolation of total RNA, using the Magtration nucleic acid purification kit (Precision System Science, Chiba, Japan). Reverse transcription was performed using the Transcriptor First Strand cDNA Synthesis Kit (Roche Applied Science, Basel, Switzerland).

### Real-time PCR

Real-time PCR was conducted using TaqMan technology, according to the manufacturer’s instructions (Applied Biosystems, Japan), in a Roche LightCycler 96 System (Roche Applied Science). The following PCR conditions were applied: one cycle of preincubation at 95°C for 600 s, and subsequent 45 cycles at 95°C for 20 s and 60°C for 40 s in a two-step amplification. TaqMan primer/probe sets (Applied Biosystems) were obtained for the following genes: glyceraldehyde 3-phosphate dehydrogenase (*GAPDH*) (Hs02758991_g1); interleukin 6 (*IL6*) (Hs00985639_m1); *IL17A* (Hs00174383_m1); tumor growth factor B1 (*TGFB1*) (Hs00998133_m1); *TGFB2* (Hs00234244_m1); tumor necrosis factor (*TNF*) (Hs99999043_m1); interferon-γ (*IFNG*) (Hs00989291_m1); vascular endothelial growth factor A (*VEGFA*) (Hs00900055_m1); chemokine C-C motif ligand 2 (*CCL2*) (Hs00234140_m1); *CCL5* (Hs00174575_m1); chemokine C-X-C motif ligand 1 (*CXCL1*) (Hs00236937_m1); *CXCL10* (Hs01124251_g1); signal transducer and activator of transcription 3 (*STAT3*) (Hs00374280_m1); v-rel avian reticuloendotheliosis viral oncogene homolog A (*RELA*) (Hs00153294_m1); retinoic acid receptor-related orphan receptor gamma (*RORC*) (Hs01076122_m1); T-box protein 21 (*TBX21*) (Hs00203436_m1); actin, alpha 1, skeletal muscle (*ACTA1*) (Hs00559403_m1); glial fibrillary acidic protein (*GFAP*) (Hs00909233_m1); tenascin C (*TNC*) (Hs01115665_m1); and periostin (*POSTN*) (Hs01566734_m1). Gene expression levels were analyzed using the relative quantification method. Briefly, Ct values obtained for each gene were normalized to *GAPDH* level, in order to obtain ΔCt values. MH control samples were used as reference samples, to obtain the ΔΔCt values. The data are presented as the fold-change relative to the gene expression levels in MH group, using the 2^-ΔΔCt^ formula.

### Statistical analyses

All statistical analyses were performed using the JMP 9 software (SAS Institute Inc., Cary, NC, USA). The obtained data are presented as mean ± standard deviation (SD). For normally and equally distributed data, gene expression levels between groups were compared using *t*-test. For normally and unequally distributed data, Welch’s *t*-test was used. Fisher’s exact test was performed for the categorical data, because of the gender classification of patients with two investigated diseases (MH or iERM). The correlation between these two parameters and cytokine, transcription factor, cytoskeletal, or extracellular matrix gene expressions was determined using the Spearman’s correlation coefficient. *P* < 0.05 was considered a statistically significant difference. For the analyses of the correlation between genes, ΔCt values obtained for each sample were used.

## Results

The demographic characteristics of the patients belonging to the two disease groups (MH and iERM) are presented in Table 1. Significant difference in age, but no significant differences in gender and surgery time were observed between the MH and iERM groups. All investigated eyes had cataract with nuclear opacity of Emery grade 2, and they underwent phacoemulsification and aspiration. Among the eyes with MH, one eye was classified as stage 3 and four eyes were classified as stage 2, according to the Gass Classification System [10]. The average hole diameter was 337 ± 193 μm.

**Table 1:** Demographics of Patients Included in Macular Hole and Idiopathic Epiretinal Membrane Groups.

	Macular hole eyes	Epiretinal membrane eyes	*P* value
Study population, number (%)	5 (25)	15 (75)	
Age, mean (SD), years	61.8 (5.84)	71.5 (6.59)	< 0.05^†^
Gender, number			0.61^‡^
Male, 7	1	6	
Female, 13	4	9	
Operation time, mean (SD), min	61 (14)	61 (12)	0.98^†^

SD, standard deviation.

^†^*t*-test or,

^‡^Fisher’s exact test were used for the comparisons between the two groups.

### Expression levels of cytokine, transcription factor, cytoskeletal, and extracellular matrix genes

The levels of genes encoding 11 cytokines, four transcription factors, two cytoskeletal, and two extracellular matrix proteins, measured in the solid component of the irrigation solution are summarized in Table 2. *IL6*, *TGFB2*, *VEGFA*, *CXCL1*, *RELA*, *GFAP*, and *TNC* were significantly upregulated (4.4-, 7.9-, 8.7-, 3.8-, 1.6-, 2.8-, and 5.8-fold, respectively; *P* < 0.05) in the iERM eyes, compared with MH eyes. *IL17A* and *ACTA1* were not detected in the irrigation solution obtained from either iERM or MH eyes. The levels of *TGFB1*, *TNF*, *IFNG*, *CCL2*, *CCL5*, *CXCL10*, *STAT3*, *RORC*, *TBX21*, and *POSTN* did not differ between the eyes with iERM and MH.

**Table 2:** Expression Levels of Cytokines and Other Investigated Genes in the Solid Component of the Irrigation Solution.

Gene	Comparative Ct method				
	ΔCt (SD)		ΔΔCt (SD)	Fold-change value; 2^-ΔΔCt^	*P* value
	Epiretinal membrane	Macular hole			
*IL6*	8.63 (1.62)	10.8 (1.48)	-1.96 (0.136)	4.4	< 0.05^‡^
*IL17A*	N.D.	N.D.	N/A	N/A	N/A
*TGFB1*	3.66 (1.10)	3.57 (0.927)	-0.0112 (0.254)	0.94	0.83^†^
*TGFB2*	4.65 (0.961)	7.63 (2.19)	-3.26 (1.48)	7.9	< 0.001^‡^
*TNF*	8.81 (1.46)	8.75 (1.11)	0.330(0.662)	0.96	0.90^†^
*IFNG*	16.5 (1.48)	15.3 (3.12)	0.616 (1.99)	0.43	0.49^‡^
*VEGFA*	3.45 (1.03)	6.58 (1.20)	-3.27 (0.317)	8.7	< 0.001^‡^
*CCL2*	4.82 (1.45)	4.02 (1.75)	0.919 (0.302)	0.57	0.42^†^
*CCL5*	9.88 (1.36)	8.90 (3.00)	0.658 (1.53)	0.51	0.20^‡^
*CXCL1*	6.87 (1.71)	8.80 (1.14)	-2.26 (0.0509)	3.8	< 0.01^‡^
*CXCL10*	12.0 (1.86)	9.32 (1.26)	2.88 (0.766)	0.16	0.29^‡^
*RELA*	7.83 (0.783)	8.47 (0.321)	-0.980 (0.449)	1.6	< 0.05^‡^
*STAT3*	6.82 (0.585)	7.31 (0.805)	-0.393 (0.104)	1.4	0.25^†^
*RORC*	13.0 (1.33)	12.7 (1.25)	-0.0011 (0.339)	0.83	0.99^†^
*TBX21*	12.1 (1.67)	11.4 (2.63)	0.235 (1.02)	0.61	0.41^†^
*ACTA1*	N.D.	N.D.	N/A	N/A	N/A
*GFAP*	0.658 (1.24)	2.14 (1.04)	-1.46 (0.0919)	2.8	< 0.05^‡^
*TNC*	2.91 (2.10)	5.45 (0.959)	-2.96 (0.308)	5.8	< 0.005^‡^
*POSTN*	14.8 (2.29)	8.47 (0.740)^#^	5.23 (1.15)	0.013	0.26^‡^

N.D., not detected; N/A, not applicable; *IL*, interleukin; *TGF*, tumor growth factor; *TNF*, tumor necrosis factor; *IFNG*, interferon-γ; *VEGF*, vascular endothelial growth factor; *CCL*, chemokine C-C motif ligand; *CXCL*, chemokine C-X-C motif ligand; *RELA*, v-rel avian reticuloendotheliosis viral oncogene homolog A; *STAT*, signal transducer and activator of transcription; *RORC*, retinoic acid receptor-related orphan receptor gamma; *TBX*, T-box protein; *ACTA1*, actin, alpha 1, skeletal muscle; *GFAP*, glial fibrillary acidic protein; *TNC*, tenascin C; *POSTN*, periostin.

^†^ *t*-test or,

^‡^ Welch’s *t*-test were used for the comparisons between the two groups.

^#^ Undetectable in three out of five eyes with macular hole.

### Correlation between the gene expression and visual acuity

A univariate analysis of correlation (Spearman’s correlation coefficient) was performed for the seven genes upregulated in the iERM eyes (*IL6*, *TGFB2*, *VEGFA*, *CXCL1*, *RELA*, *GFAP*, and *TNC*) and the preoperative visual acuity (assessed using a logMAR chart) of the eyes with MH or iERM. No significant positive correlations were observed (Table 3). An additional Spearman’s correlation coefficient analysis was performed in order to compare the expression of these seven genes between the eyes with iERM. As shown in Table 4, strong positive correlations between *TGFB2* and *IL6*, *CXCL1*, or *RELA*, *VEGFA* and *GFAP* or *TNC*, and *GFAP* and *TNC* were detected. We showed scatterplots of raw data (S1 Fig), and presented *p* value acquired by Spearman’s correlation coefficient analysis.

**Table 3:** Spearman’s Correlation Coefficients between the Seven Upregulated Genes and Preoperative Visual Acuity of Eyes with Idiopathic Epiretinal Membrane.

	Spearman correlation coefficients, ρ (*P* value)						
	*IL6*	*TGFB2*	*VEGFA*	*CXCL1*	*RELA*	*GFAP*	*TNC*
Preoperative visual acuity	-0.153 (0.62)	0.178 (0.53)	-0.166 (0.55)	0.195 (0.49)	0.0911 (0.75)	0.102 (0.73)	-0.186 (0.51)

*IL*, interleukin; *TGF*, tumor growth factor; *VEGF*, vascular endothelial growth factor; *CXCL*, chemokine C-X-C motif ligand; *RELA*, v-rel avian reticuloendotheliosis viral oncogene homolog A; *GFAP*, glial fibrillary acidic protein; *TNC*, tenascin C.

**Table 4:** Spearman’s Correlation Coefficient Analysis of the Expression Levels of Seven Genes in Eyes with Idiopathic Epiretinal Membrane.

	Spearman’s correlation coefficients	
	ρ	*P* value
*IL6* vs. *TGFB2*	0.762	< 0.005
*IL6* vs. *VEGFA*	0.110	0.72
*IL6* vs. *CXCL1*	0.423	0.15
*IL6* vs. *RELA*	0.300	0.31
*IL6* vs. *GFAP*	0.189	0.56
*IL6* vs. *TNC*	0.209	0.49
*TGFB2* vs. *VEGFA*	0.0519	0.85
*TGFB2* vs. *CXCL1*	0.648	< 0.01
*TGFB2* vs. *RELA*	0.597	< 0.05
*TGFB2* vs. *GFAP*	0.260	0.37
*TGFB2* vs. *TNC*	0.385	0.16
*VEGFA* vs. *CXCL1*	-0.281	0.31
*VEGFA* vs. *RELA*	-0.0680	0.81
*VEGFA* vs. *GFAP*	0.624	< 0.05
*VEGFA* vs. *TNC*	0.686	< 0.005
*CXCL1* vs. *RELA*	0.476	0.073
*CXCL1* vs. *GFAP*	-0.0882	0.76
*CXCL1* vs. *TNC*	0.129	0.65
*RELA* vs. *GFAP*	0.199	0.50
*RELA* vs. *TNC*	0.335	0.22
*GFAP* vs. *TNC*	0.705	< 0.005

*IL*, interleukin; *TGF*, tumor growth factor; *VEGF*, vascular endothelial growth factor; *CXCL*, chemokine C-X-C motif ligand; *RELA*, v-rel avian reticuloendotheliosis viral oncogene homolog A; *GFAP*, glial fibrillary acidic protein; *TNC*, tenascin C.

## Discussion

Retinal pigment epithelial cells, glial cells, endothelial cells, myofibroblast-like cells, macrophages, fibrocytes, fibrous astrocytes, as well as trophic and transcription factors may be associated with the formation of epiretinal membranes [1,3,8,9,11,12]. Several studies suggested that these cells form a scar-like thin layer as an immune response to protect the retina [3,4,13,14]. Growth factors, cytokines, and extracellular matrix proteins may be involved in the intracellular signaling and histologic changes during the ERM formation process as well [5–8,12,13,15].

Here, the expression levels of four cytokine-encoding genes (*IL6*, *TGFB2*, *VEGFA*, and *CXCL1*) and three other genes (*RELA*, *GFAP*, and *TNC*) were shown to be significantly higher in the iERM eyes than in the MH control eyes. The upregulation of these genes may be associated with the iERM formation. Even though the number of females included in our study was 1/3 higher than the number of males, the genes shown to be upregulated did not significantly differ between them (S2 Table).

Only *RELA* gene expression was shown to be positively correlated with age (S1 Fig). The relationship of advancing age and ERMs has been reported previously [2]. RELA is a subunit of the nuclear factor kappa B (NF-κB) heterodimer, which is a transcription factor that can be activated by various stimuli, such as hypoxia, bacterial and viral infections, and proinflammatory cytokines, including TNF-α and IL1β, and which plays significant roles in immune response, inflammation, apoptosis, and cellular proliferation [16,17]. NF-κB and IL8, an angiogenic factor, were found to be expressed in the glial and vascular endothelial cells, and their expression levels were associated with ERM formation [12]. This suggests that the advanced age may be associated with the upregulation of *RELA* in glial and endothelial cells, and, subsequently, ERM formation.

Furthermore, positive correlations between *TGFB2* and *IL6*, *CXCL1* or *RELA* were determined. *TGFB2* upregulation was reported in eyes with iERM, proliferative diabetic retinopathy (PDR), and proliferative vitreoretinopathy (PVR), and it is associated with intraocular fibrosis [5,7,13,15]. TGFB2 was shown to induce the transformation of the retinal pigment epithelial (RPE) cells, hyalocytes, and myofibroblastic cells [5,7,13]. Minchiotti *et al*. [8] showed that the expression of *TGFB1* is elevated in ERM vitreous. However, the expression of *TGFB2*, but not *TGFB1*, was found to be higher in iERM samples than in MH samples in our study. Iannetti *et al*. [5] also reported that the correlation between *TGFB2* expression and iERM is more significant than between *TGFB1* expression and iERM development. Kohno *et al*. [7] showed a strong contractile activity of collagen gels and αSMA (ACTA1) overexpression in the hyalocytes in the presence of TGFB2. They also reported that iERM was composed of αSMA- and GFAP-positive cells in the ERM foci and periphery, respectively. This suggests that *ACTA1* (gene encoding αSMA) expression level may be too low to be possible to detect it by real-time PCR in the irrigation solution. IL6, a pro-inflammatory factor, was previously reported to be involved in the activation of Müller glial cells via microglia [18] and in the epiretinal formation in eyes, particularly those with PDR and PVR [19]. To the best of our knowledge, the correlation between *CXCL1* expression and iERM *in vivo* has not been reported previously. However, *CXCL1* expression may be associated with iERM formation as CXCL1 is involved in angiogenesis, inflammation, and wound healing process [20,21]. Iwona *et al*. [22] reported that RPE-stimulated Müller glial cells react to recombinant human CXCL1.

Previous reports have showed the association between GFAP-expressing glial cells (Müller cells or astrocytes) and iERM formation [1,2,4,6,7,12–14,23]. Additionally, retinal glial cells were reported to produce VEGF [24]. In our study, a significant positive correlation was observed between *VEGFA* and *GFAP* gene expression levels in the iERM eyes (ρ = 0.624, *P* < 0.05; Table 4). *GFAP*-positive cells may induce the proliferation and contraction of other cells [25,26]. Strong positive correlations were also observed between *VEGFA* and *TNC* expression levels (ρ = 0.686, *P* < 0.005; Table 4), and between *GFAP* and *TNC* expression levels (ρ = 0.624, *P* < 0.05; Table 4). The *TNC* gene encodes the extracellular protein tenascin C, which is involved in wound healing, inflammation, and the sprouting of endothelial cells during angiogenesis have been described [27].

**Fig 1:**
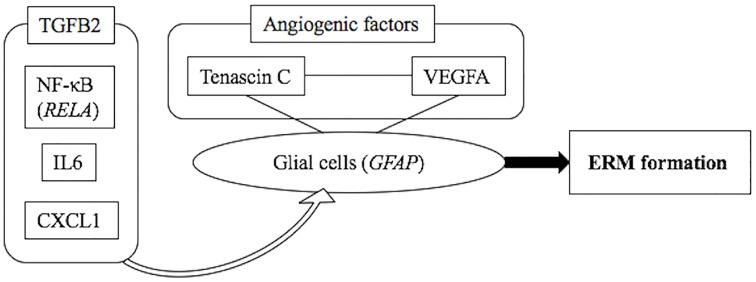
A model illustrating the putative relationship between the seven genes shown to be upregulated in iERM eyes.

In summary, *TGFB2* expression was shown to be positively correlated with *IL6*, *CXCL1*, and *RELA* expression levels. In addition, *VEGFA*, *GFAP*, and *TNC* were themselves correlated (Table 4). The data shown in Table 4 and previous studies support the hypothesis that NF-κB (*RELA*), IL6, and *CXCL1* are activated upon stimulation (*e*.*g*., by a posterior vitreous detachment during aging) [22] in a TGFB2-rich environment [5,7,16,17] Subsequently, NF-κB induces glial cells [12] to produce VEGFA [23], which, along with tenascin C, promotes the endothelial cell growth [26,27]. Furthermore, NF-κB promotes *CXCL1* transcription [20], stimulating glial cells further [21]. Taken together, the factors involved in angiogenesis and wound healing processes may be associated with the iERM formation (Fig 1).

Additionally, we showed that the expression of T-cell cytokines and other T-cell associated factors, including *IL17A*, *IFNG*, *RORC*, and *TBX21*, did not correlate with iERM formation. Further investigations are needed to elucidate iERM pathogenesis, as the low number of subjects enrolled and a significant difference in the age of the subjects between two groups in this study represent the limitations of this study. Additionally, protein expression and the activation of signaling pathways should be further analyzed as well.

Three genes (*IL6*, *CXCL1*, and *RELA*) were positively correlated with *TGFB2*, while other three genes (*VEGFA*, *GFAP*, and *TNC*) were themselves correlated. Based on these findings, we hypothesized that TGFB2 and three factors (IL6, CXCL1, and NF-κB) might stimulate GFAP-positive cells related to fibrosis, and angiogenic factors (VEGFA and Tenascin C) can augment iERM formation.

TGF, tumor growth factor; NF-κB, nuclear factor kappa B; *RELA*, v-rel avian reticuloendotheliosis viral oncogene homolog A; IL, interleukin; *CXCL*, chemokine C-X-C motif ligand; VEGF, vascular endothelial growth factor; *GFAP*, glial fibrillary acidic protein; ERM, epiretinal membrane.

## Conclusions

We evaluated gene expression levels in the solid component of the irrigation solution sample collected during pars plana vitrectomies of eyes with MH or iERM. Previous studies utilized ELISA, immunoblotting, or traditional RT-PCR to compare the level of several cytokines and other factors in the vitreous humor or membranes of patients with ERM [5–9]. To the best of our knowledge, this is the first time real-time PCR analysis has been employed to assess the expression levels of cytokines and/or other genes in the irrigation solution samples collected during vitrectomy.

The factors associated with angiogenesis and wound healing processes were more upregurated in the eyes with iERM than with MH, and the factors associated with T-cell did not seem to affect ERM formation. This study may help elucidate the pathogenesis of iERM, but it may also represent a foundation for future studies of cytokines and other molecules involved in the development of secondary ERM.

## Supporting Information

S1 Fig Correlation Between the Expression Levels of the Upregulated Genes and Age or Preoperative Visual Acuity in Idiopathic Epiretinal Membrane Group. *p* values acquired by Spearman’s correlation coefficient analysis were shown in the scatterplots.
(TIFF)

S1 Table The Obtained Ct Value of the Investigated Genes in the Solid Component of the Irrigation Solution. (TIFF)

S2 Table The Differences in the Expression Levels of the Upregulated Genes between Males and Females with Idiopathic Epiretinal Membrane. ^†^*t*-test was used for the comparisons between male and female in the seven upregulated genes or age.
(TIFF)
