# Supplementary figures and images for: Gene Expression Analysis of the Irrigation Solution Samples Collected during Vitrectomy for Idiopathic Epiretinal Membrane
Source: PLoS One. 2016 Oct 13;11(10):e0164355. doi: 10.1371/journal.pone.0164355 (PMC5063277; doi:10.1371/journal.pone.0164355)

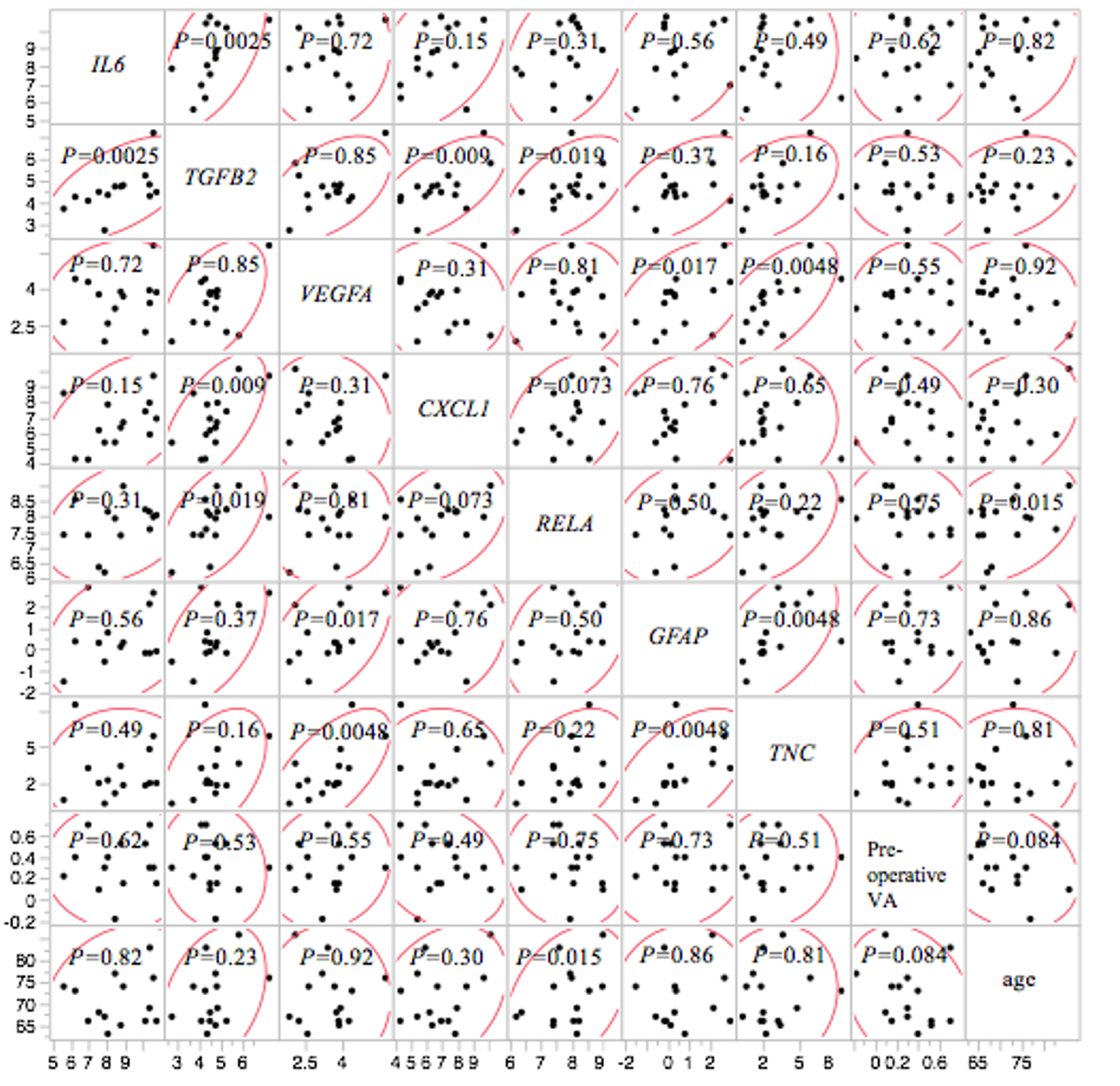

Supplement: S1 Fig — p values acquired by Spearman’s correlation coefficient analysis were shown in the scatterplots. (TIFF) [file pone.0164355.s001.tiff]

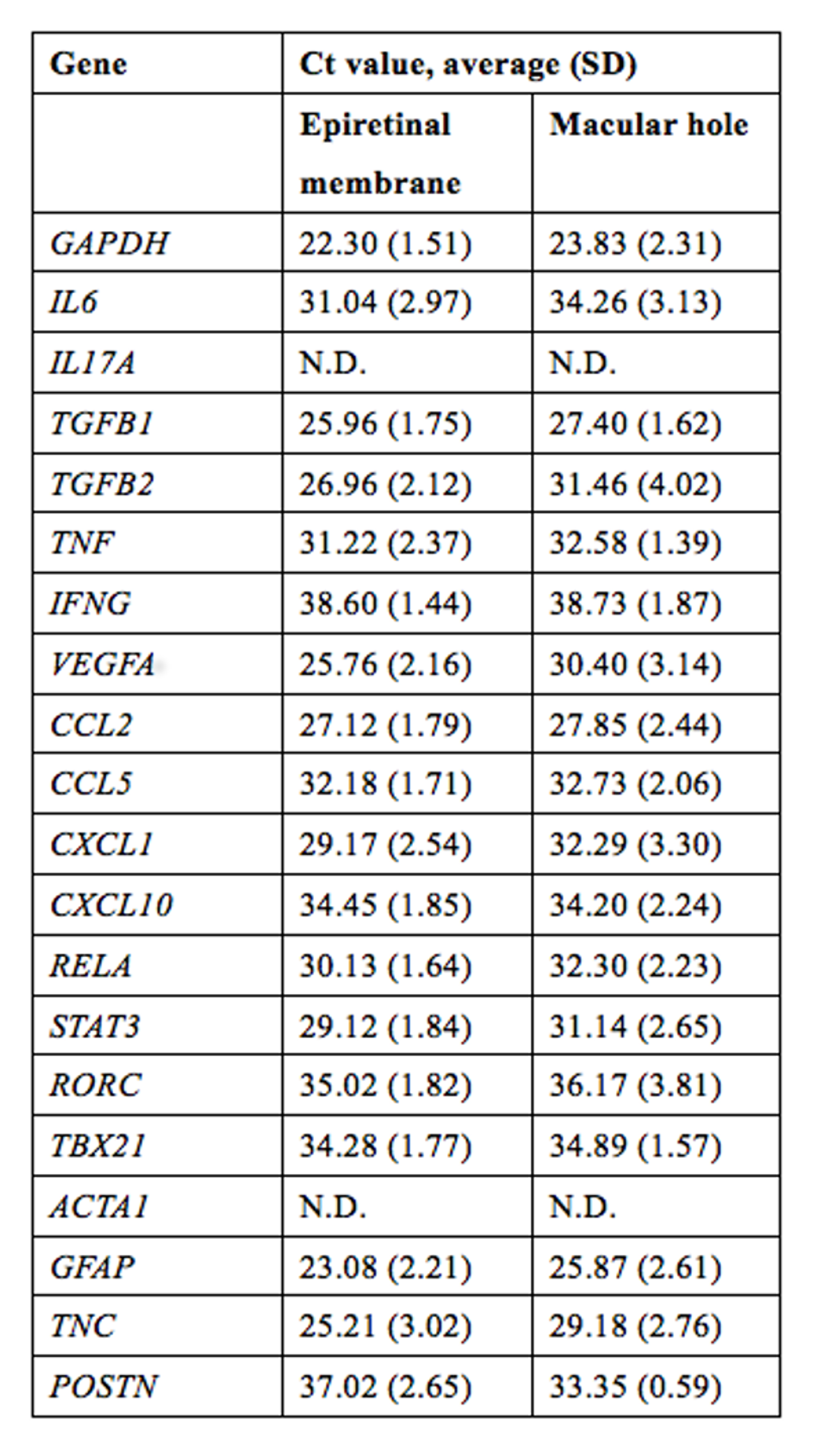

Supplement: S1 Table — (TIFF) [file pone.0164355.s002.tiff]

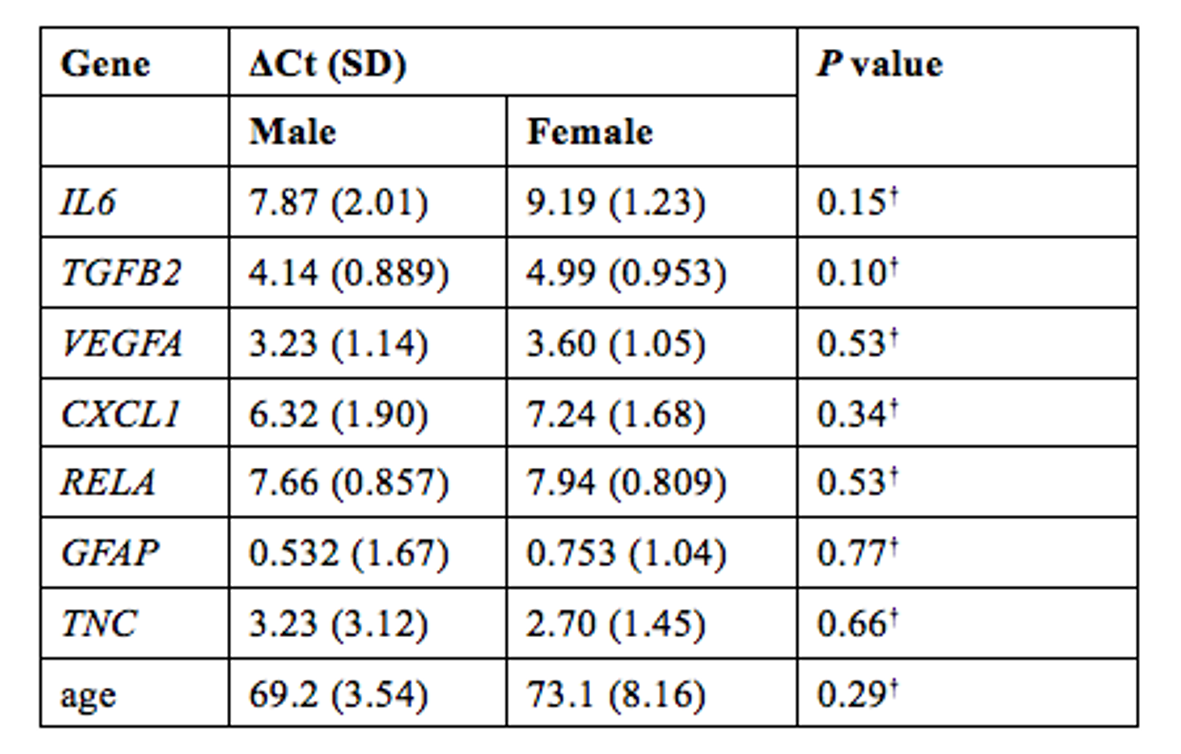

Supplement: S2 Table — †t-test was used for the comparisons between male and female in the seven upregulated genes or age. (TIFF) [file pone.0164355.s003.tiff]
